# Autonomic Nervous System Response to Psychosocial Stress in Anorexia Nervosa: A Cross-Sectional and Controlled Study

**DOI:** 10.3389/fpsyg.2021.649848

**Published:** 2021-03-17

**Authors:** Ileana Schmalbach, Benedict Herhaus, Sebastian Pässler, Sarah Runst, Hendrik Berth, Silvia Wolff, Bjarne Schmalbach, Katja Petrowski

**Affiliations:** ^1^Department of Medical Psychology and Medical Sociology, University Medical Center of the Johannes-Gutenberg University Mainz, Mainz, Germany; ^2^Division of Psychological and Social Medicine and Developmental Neurosciences, Research Group Applied Medical Psychology and Medical Sociology, Carl Gustav Carus Faculty of Medicine, Technische Universität Dresden, Dresden, Germany; ^3^Department of Psychotherapy and Psychosomatic Medicine, University Hospital Carl Gustav Carus Dresden, Dresden, Germany; ^4^Abteilung für Innere Medizin III, Universitätsklinikum Carl Gustav Carus an der Technischen Universität Dresden, Dresden, Germany

**Keywords:** eating disorders, anorexia nervosa, trier social stress test, heart rate variability, laboratory stress induction, HRV in AN

## Abstract

To foster understanding in the psychopathology of patients with anorexia nervosa (P_AN_) at the psychological and physiological level, standardized experimental studies on reliable biomarkers are needed, especially due to the lack of disorder-specific samples. To this end, the autonomic nervous system (ANS) response to a psychosocial stressor was investigated in *n* = 19 P_AN_ (BMI: 18.7 ± 3.3 kg/m^2^), age, and gender-matched to *n* = 19 healthy controls (HC; BMI: 24.23 ± 3.0 kg/m^2^). For this purpose, heart rate (HR) and heart rate variability (HRV) parameters were assessed in a cross-sectional study design under two experimental conditions: (1) rest and (2) stress (Trier Social Stress Test). In addition, psychological indicators of stress were assessed. An 2 × 2 × 8 ANOVA demonstrated similar HR and HRV patterns (except LF-HRV) between P_AN_ and HC at rest. Under stress, P_AN_ (vs. HC) demonstrated a blunted HR [condition^*^time^*^group: *F*_(2.91, 104.98)_ = 9.326, *p* = 0.000, η^2^ = 0.206] and an attenuated HRV response (reduced SNS/PNS reactivity). Significant effects of stress appraisal (SA) and BMI on HRV-reactivity were revealed. SA on SDNN = Condition^*^time^*^SA = *F*_(4.12, 140.15)_ = 2.676, *p* = 0.033, η^2^ = 0.073. BMI on LF/HF-Ratio = Condition^*^time^*^BMI = *F*_(3.53, 60.16)_ = 3.339, *p* = 0.019, η^2^ = 0.164. Psychological indices suggested higher levels of chronic and appraised stress in P_AN_ relative to HC. Additional analyses demonstrated that ED-symptoms are highly correlated with the latter constructs, as well as with psychological burden, but not with weight. Further, it was shown that abnormalities in reactivity persisted despite normalized ANS activity. Overall, we suggested that besides weight recovery, improvement in stress appraisal could be beneficial for cardiac health. In this light, a combination of therapy (e.g., development and activation of coping skills, cognitive reappraisal) and biofeedback training may improve treatment outcomes and regulate stress reactivity.

## Introduction

Heart Rate Variability (HRV) is a convenient method to assess Autonomic Nervous System (ANS) functionality: It reflects the contribution of parasympathetic (PNS), and sympathetic (SNS) activity, and acts as a biomarker for health, and adaptive stress behavior in social contexts (Marques et al., 2010; Young and Benton, 2015; Shaffer and Ginsberg, 2017; Lischke et al., 2018). A balanced HRV is related to positive health outcomes, while a low to physical and psychological morbidity (Thayer et al., 2010; Young and Benton, 2018) as in the case of patients with anorexia nervosa (P_AN_). Anorexia nervosa (AN) is an eating disorder (ED), typically characterized by an exaggerated fear of weight gain, constant weight and shape concerns in spite of being underweight (BMI < 17.5 kg/m^2^; (DSM-5, 2013; Seidel et al., 2018)). P_AN_ face several medical risks and exhibit a wide range of ANS dysfunctions (e.g., cardiovascular irregularities), registering at least five times greater rate of death than in the general population (Katzman, 2005; Arcelus et al., 2011; Keshaviah et al., 2014; Sachs et al., 2016). Treatment outcomes are poor (Harbottle et al., 2008; Watson and Bulik, 2013; Murray et al., 2019) and many patients still report decreased well-being and low quality of life (Jenkins et al., 2011; Tomba et al., 2017). Thus, innovative treatments are urgently needed. The role of psychosocial stress (e.g., social evaluation, exclusion, contexts related to performance; Kirschbaum et al., 1993; Pruessner et al., 2003; Dickerson and Kemeny, 2004) in the onset and maintenance of AN, as well as its effects on the ANS functionality has been frequently thematized (Caglar-Nazali et al., 2014; Monteleone et al., 2018; Wierenga et al., 2018; Young and Benton, 2018). Almost 90% of P_AN_ exhibit cardiac abnormalities (Giovinazzo et al., 2019) with a high incidence of bradycardia (i.e., low resting HR <50/min; Mazurak et al., 2011; Portilla, 2011; Gibson et al., 2020). However, experimental studies on their ANS response are underrepresented, especially in the context of psychosocial stress. Additionally, empirical evidence on ANS reactivity is still inconsistent. In terms of HRV tone (at rest), three different patterns are reported in P_AN_: some studies found increased parameters (Roche et al., 2004; Bär et al., 2006), while others decreased (Platisa et al., 2006; Lachish et al., 2009), and a third group of papers unveiled comparable results between patients and healthy controls (HC; Murialdo et al., 2007; Vigo et al., 2008). Nevertheless, a general PNS/SNS imbalance with a trend in parasympathetic hyperactivity (Mazurak et al., 2011; Jacoangeli et al., 2013; Bomba et al., 2014) and sympathetic hypoactivity (at rest; Mazurak et al., 2011) has been constantly observed in P_AN_. Concerning heart rate (HR) reactivity, some researchers observed a blunted response in patients (Monteleone et al., 2011, 2018; Het et al., 2015, 2020) compared to healthy individuals, while others found a comparable reactivity between both groups (Vocks et al., 2008; Het et al., 2020). Regarding the cardiovascular reactivity as indexed by HRV, patients demonstrated a pronounced PNS reactivity (lower HR + stronger HF-HRV) and a SNS-hyporeactivity as indexed by salivary Alpha-Amylase (sAA; Het et al., 2015, 2020). Although, the latter was not supported by LF-HRV, since it was comparable in patients and controls (Het et al., 2015). Still, HRV analyses were not specific to P_AN_, but to an ED-mixed sample. Indeed, when studying a disorder-specific sample of P_AN_, Rommel et al. (2015) demonstrated an attenuated parasympathetic response after stress induction (via 80 s. film). Other HRV parameters, rather than a low HF-HRV were not reported, leaving the role of the SNS unknown. These inconsistencies can be explained by differences in methodological procedures (e.g., unstandardized stressors), whereby the degree of ANS activation is not clear. Other influential factors are the diverse methods to analyze (e.g., WT, wavelet transformation; Rommel et al., 2015) and report ANS-parameters (e.g., sAA, either LF-HRV, or HF-HRV values) providing either a reliable nor a full picture of the ANS functionality (Bosch et al., 2011). Further, heterogeneity in patient population (e.g., mixed ED-groups) limits the comparability of results and small sample sizes make findings susceptible to random effects. Researches recommended to investigate these results in the light of ED-specific samples (Vocks et al., 2008; Het et al., 2020) due to differences in their stress response (Monteleone et al., 2011; Peschel et al., 2016). From this perspective, there is a need to clarify the ANS response in P_AN._ In general, experimental evidence on the ANS reactivity to psychosocial stress in P_AN_ is scarce and ambiguous. Considering this background, the purpose of the present study is to expand P_AN_-specific data and examine HRV parameters (see Table 1) under controlled conditions. To this end, we implemented a psychosocial and a standardized stressor (i.e., Trier Social Stress Test -TSST; Kirschbaum et al., 1993).

**Table 1:** Metrics of heart rate variability.

**The power spectrum analysis of the HRV evaluates the quantitative contribution of time (RMSSD = Square root of the mean squared differences of successive NN interval; SDNN, Standard deviation normal to normal) and frequency domains (HF, high frequent and LF, low frequent LF) to the total variance (power) of heart rate (HR)**.
**• RMSSD includes sympathetic influences and is associated to HF power, but is tendentially moderated by the PNS than SDNN (Shaffer and Ginsberg, 2017).** **• SDNN indicates a general HRV modulated by PNS and SNS branches. Mazurak et al. (2011) added, that it also records endocrine and thermoregulatory processes.** **• HF is a marker of the parasympathetic vagal tone being respiration dependent (Force, 1996; Stein and Kleiger, 1999; Shaffer and Ginsberg, 2017).** **• LF signals activity in both systems: PNS and SNS (Cohen et al., 2000; Shaffer and Ginsberg, 2017).** **• The LF/HF-ratio represents the balance between PNS and SNS activity. According to this model, a low LF/HF ratio stands for PNS dominance while a high ratio reflects SNS overactivation (Mazurak et al., 2011; Shaffer and Ginsberg, 2017). However, the SNS contribution to LF power varies depending on the testing context: e.g., under resting conditions, it shows PNS and baroreflex, rather than SNS activity (Kember et al., 2001; Goldstein et al., 2011)**.

### Hypotheses

(**H**_1_) Since bradycardia is highly prevalent in P_AN_ (Mazurak et al., 2011; Gibson et al., 2020), we estimated a significantly lower baseline HR in P_AN_ than in HC. (**H**_2_) We hypothesized PNS hyperactivity and a decreased SNS in P_AN_ at rest, when compared to HC (Mazurak et al., 2011; Jacoangeli et al., 2013; Bomba et al., 2014). (**H**_3_) We expected a blunted HR response to the TSST as consistently evidenced (Monteleone et al., 2011, 2018; Het et al., 2015, Het et al., 2020). **(H**_4_**)** In consonance with studies based on the TSST (Het et al., 2015, 2020), we assumed differences in ANS reactivity between both groups, whereby P_AN_ expressed a low HRV reactivity with a **(H**_5_**)** reduced SNS and pronounced PNS-reactivity.

## Method

### Participants

In the present study *n* = 19 P_AN_ (BMI: 18.70 ± 3.30 kg/m^2^) and *n* = 19 HC (BMI: 24.23 ± 3.04 kg/m^2^) were recruited to investigate the effects of stress on the ANS. In general, participants between 18 and 65 years of age were eligible. Patients with a primary diagnosis of AN were eligible and recruited at the Polyclinic for Psychotherapy and Psychosomatic in Dresden, Germany. Patients were diagnosed with AN prior admission to an inpatient treatment and were enrolled in the study after a stabilization period, since some were artificially nourished. Consequently, some patients experienced weight restoration. Before participation, P_AN_ were re-screening based on the Structured Clinical Interview (SCID) of the Diagnostic and Statistical Manual of Mental Disorders (DSM-IV; First et al., 1997; Wittchen et al., 1997). P_AN_ with other mental disorders besides depression (e.g., post-traumatic-stress disorder, borderline personality disorder, schizophrenia), chronic illness, and medication treatment were excluded. HC were recruited through online media, newspapers, and bulletin boards at different universities, and were screened for mental or physical conditions. Only mentally and physically healthy participants were included. HC with an abnormal BMI, chronic illness, medication treatment, and stressful life events in the past 6 months were excluded. Participants who did not refrain from eating or smoking 3 h before testing were excluded too. Six participants were excluded due to substantial missing data resulting in a final sample of *N* = 38.

Every participant received an expense allowance of 50 Euro after participation on two test days—within a week. A description of all *N* =38 participants is displayed in Table 2. Ethical approval was obtained from the Ethics Committee of the Medical faculty of the Technical University of Dresden, Germany (No#EK25012013).

**Table 2:** Characteristic of the participants—Demographic variables.

	**P_**AN**_**	**HC**	***t*/*U***	***p***
Total (*N*)	19	19		
Female, *n* (%)	17 (89.5)	17 (89.5)		
Age *M (SD)*	26.05 (5.49)	24.21 (5.54)	1.03	0.31
BMI *M (SD)*	18.70 (3.30)	24.23 (3.04)	−5.36	0.000***
Contraceptives *n* (% females)	44.73 (17)	31.57 (12)	3.64	0.062
TICS *M*(SD)	23.90 (10.14)	17.47 (5.59)	2.41	0.022
Smokers, *n* (%)	13.15 (5)	2.63 (1)	3.17	0.090
Sport *(MR)*	16.50	22.50	43.50 ^(a)^	0.961
Sleep problems *(MR)*	24	15	42.00 ^(a)^	0.849

P_AN_, Participants with Anorexia nervosa; HC, Healthy Controls; M, Mean; MR, MeanRank; SD, Standard Deviation; TICS, Trier Inventory Chronic Stress; <p ≤ 0.001***, (a) Mann-Whitney-Test.

### Procedure

**Figure 1:**
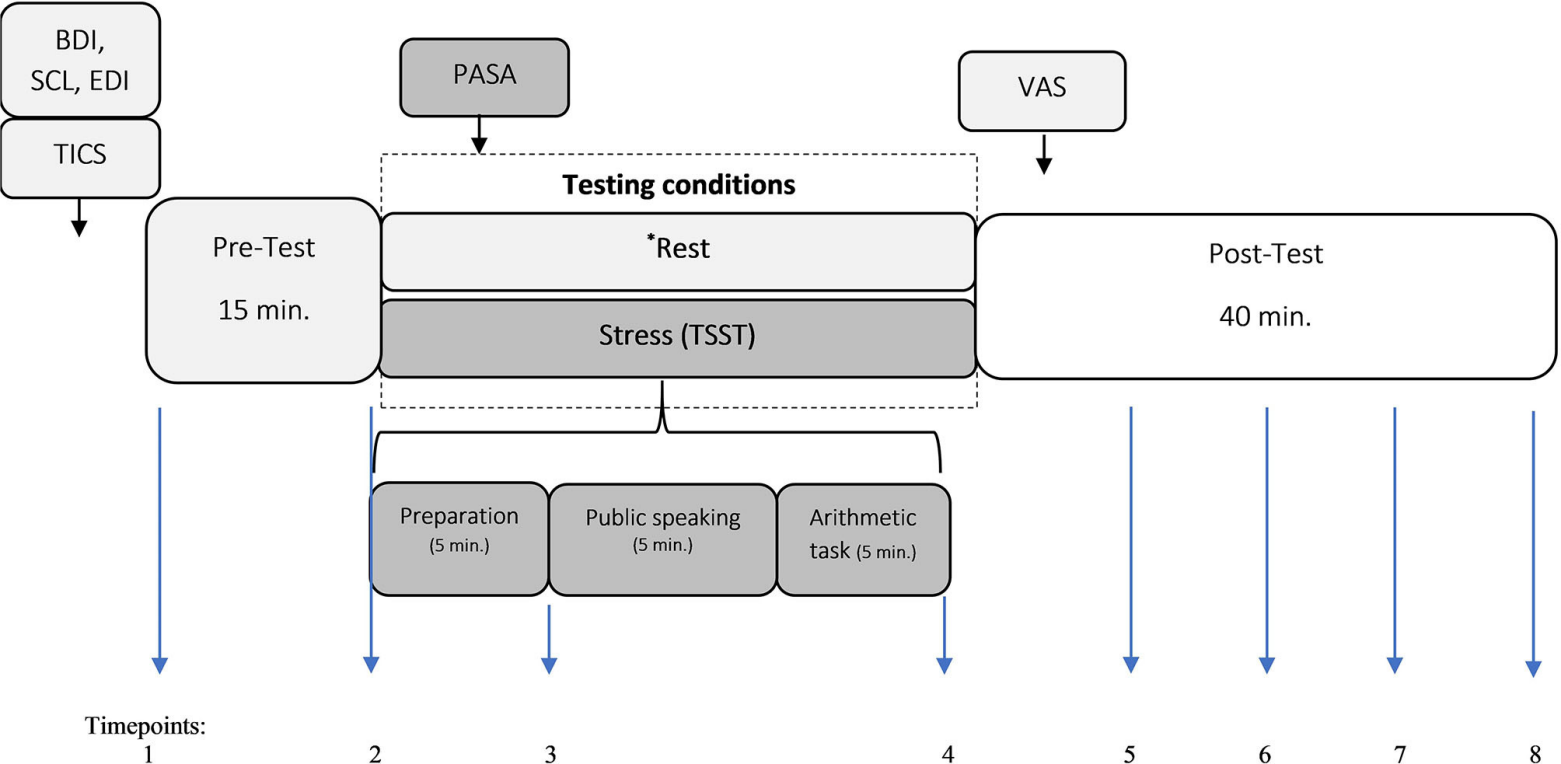
Experimental design. Trier Inventory for the assessment of Chronic Stress; PASA, Primary Appraisal Secondary Appraisal questionnaire; VAS, Visual Analog Scale; BDI, Beck Depression Inventory; SCL, Symptom Check List-K-9; EDI, Eating Disorder Inventory; TSST, Trier Social Stress Test. Measurement timepoints = 1 (Baseline), 2 (Preparation), 3 (Before public speaking), 4 (After math task), 5–8 (Recovery Phase).^*^ The measurements in the control condition were collected on equivalent timepoints.

P_AN_ and HC were scheduled on 2 days for two different experimental conditions (*stress* and *rest*) in order to minimize the probability of errors. All participants were registered between 2:00 p.m. and 4:00 p.m. and were previously requested to refrain from eating, drinking and smoking prior examination. Upon arrival, participants signed an informed consent form and filled out psychological measures for ~30 min. Thereafter, they were fitted with a long-term electrocardiogram (ECG) belt-recorder for continuous ECG recording (SysVital, Fraunhofer IPMS, Germany). The participants were *randomly* assigned either to the *stress* or to the *resting* condition: 19 participants started with the resting, while the other 19 with the stress condition. The derived HR and HRV parameters were computed over 3-min intervals during a pre-TSST resting period (15 min), during stress exposure (15 min TSST), and during a post-TSST recovery period (40 min). These data were collected over eight Measurement timepoints = 1 (Baseline), 2 (Preparation), 3 (Before public speaking), 4 (After a math task), 5–8 (Recovery Phase). The measurements in the control condition were collected on equivalent timepoints (see Figure 1). The testing started with the resting period (15 min.) and was concluded with a recovery period lasting 40 min. In the next testing day, the participants took part on the remaining condition. During the respective experimental condition every participant completed the Primary Appraisal Secondary Appraisal questionnaire (PASA; Gaab et al., 2005). At the end of the resting or stress condition, the participants rated their perceived stress by means of the visual analog scale (VAS; Flint et al., 2000). An overview of the procedure is illustrated in Figure 1.

### Experimental Conditions: Stress vs. Rest

#### Stress Condition

The Trier Social Stress Test (TSST; Kirschbaum et al., 1993; Kudielka et al., 2007) is a validated and a standardized procedure, which is world-wide recognized for its effective stress induction and activation of the ANS (Kirschbaum et al., 1993; Mohammadi et al., 2019). In short, it consists in a social evaluation with a lack of feedback, in front of a selected two-person panel and a mental arithmetic test, divided in three phases lasting 5 min each (1st: Speech preparation. 2nd: Public speaking. 3rd: Arithmetic task).

#### Resting Condition

In this experimental phase participants could read fordable literature in a quiet and secluded room.

### Heart Rate and Heart Rate Variability

The HR and HRV variables were computed by means of a three-channel ECG. The recordings were taken from all of the participants during the entire experimental conditions. A heart rate tachogram was determined from the ECG data by specifying the heart beats and evaluating the RR intervals. Subsequently, the ECG recordings were sampled with a frequency of 250 Hz, an amplitude quantification of 12 bit, and an analogous recording bandwidth with 0.5–80 Hz. Next, the heart beat spotting was computed by means of a wavelet min-max-pair based automated QRS detector (Zaunseder et al., 2008). Prior to analyses, data was filtered for artifacts and erroneous R–R interval by the Polar ProTrainer 5 (Polar, Germany). This software works with an automatic filtering method (filter power: moderate, minimum protection zone: 6 sqm). In the present study, the HRV parameters were calculated in three min. intervals. The following time (expressed in ms) and frequency (ms^2^) parameters were analyzed to specify HRV= HF, LF power, LF/HF ratio, RMSSD, and SDNN.

### Psychological Questionnaires

*Trier Inventory for the assessment of Chronic Stress* (TICS-9; Schulz et al., 2004; Petrowski et al., 2019a). The TICS-9 is a short version of the original 57-item TICS. We applied the short form to assess chronic stress during the last 3 months. This version includes nine items reflecting all dimensions of the long version (e.g., *work and social overload, pressure to perform, social tensions*). The items can be rated from 0 to 5 (0 = never, 1 = rarely, 2 = sometimes, 3 = often, 4 = very often). Higher values suggest greater stress. Satisfactory psychometrics have been evidenced in several studies, with Cronbach's Alpha ranging from α = 0.88–0.91 (Petrowski et al., 2012, 2018, 2019a).

*Primary Appraisal Secondary Appraisal questionnaire* (PASA; Gaab et al., 2005). This scale evaluates situational and disorder-specific cognitions. The primary appraisal evaluates specific situations as *threatening* or *challenging*. The second appraisal reflects perceived coping capabilities (*self-concept* and *control expectancy*). Based on these subscales, a total stress index (SI) can be calculated. It comprises 16 items using a 6-point Likert scale (1 = completely disagree to 6 = completely agree) and exhibits reasonable psychometric properties (Cronbach's alpha = 0.61–0.83; Gaab et al., 2005; Carpenter, 2016).

*Visual Analog Scale* (VAS; Flint et al., 2000). The VAS is a widespread scale, due to its economy and reliability to evaluate stress. With this tool, our participants rated their subjective perception of stress in each experimental condition. Values ranged between no stress to severe stress (0–100; Lesage et al., 2012).

*Symptom-Check-List-9* (SCL-K-9; Klaghofer and Brähler, 2001). This scale is a short version of the original Symptom-Checklist-90-Revised (Franke and Derogatis, 2002) which evaluates general psychopathology (e.g., *somatic symptoms, interpersonal sensitivity, anxiety*). It also provides a global severity index (GSI) as an indicator of overall psychological distress; whereby higher scores indicate higher levels of psychopathological distress and greater symptom severity. For the purpose of the present study, we only reported GSI-values. The items can be rated on a 5-point Likert scale (0 = never to 4 = very often). The sum score is calculated by addition of all item scores. The scale demonstrates satisfactory psychometric properties (α = 0.83–0.87; Klaghofer and Brähler, 2001; Prinz et al., 2013; Sereda and Dembitskyi, 2016; Petrowski et al., 2019b). Normative percentile values specific to age and gender are reported by (Petrowski et al., 2019b).

*Beck Depression Inventory* (BDI; Beck et al., 1961; Hautzinger et al., 1994). The BDI is a self-report questionnaire with 21 items that can be rated from 0 to 3 (0–63). The total score is calculated by addition of the items. Higher scores indicate greater symptom severity. Cut-off values are established as follow: <14 = normal, 14–19 = mild depression, 20–28 = moderate depression, 29–63 = severe depression. Its psychometric properties are satisfactory (α = 0.89–0.94.; Beck et al., 1996; Kühner et al., 2007).

*Eating Disorder Inventory* (EDI; Garner et al., 1983; Thiel and Paul, 1988). The EDI measures symptoms and attitudes relevant to pathological eating behavior in 64 items comprised in 8 dimensions (e.g., *Drive for Thinness, Body Dissatisfaction, Interpersonal Distrust*). The items are evaluated on a 6-point rating scale (0 = never to 5 = always). The psychometric properties are satisfactory (α = 0.72–0.92; Thiel and Paul, 1988; Dinkel et al., 2005). In the present study, the evaluation of the EDI-score was based on the EDI-scores provided by (Kappel et al., 2012).

### Statistical Analyses

All statistical analyses were performed with the Statistical Package for the Social Sciences (SPSS version 24.0). The sphericity hypothesis was verified using the Mauchly test and Greenhouse–Geisser adjusted *p*-values are reported when necessary. The optimum statistical sample size was calculated with the G^*^power program (version: 3.1.9.2.). Based on a medium effect size of Cohen's *f* = 0.25, two groups (P_AN_ and HC), *n* = 8 repetitions, a significant level of *p* = 0.05, power of 80% (1–β = 0.80), and after Bonferroni-correction, a total sample size of *n* = 19 for within-subjects factor and *n* = 38 for between-subjects factor was needed. Mean differences between P_AN_ and HC in demographic characteristics (e.g., sport activity, cigarette smoking) and psychological measures, were specified by independent *t*-tests and non-parametric tests (i.e., Mann-Whitney). A two-way ANOVA for repeated measures with the between-factor *group* (P_AN_ vs. HC) and the within-factor for *condition* (rest vs. stress) and *time* (8×) was performed to calculate main or interaction effects in the parameters of interest (HR, HRV) in response to the experimental interventions. Additional *t*-tests were performed to specify time-independent differences between the experimental groups in HR and HRV parameters.

*Additional analyses*. Since the main topic of the present study refers to ANS reactivity to a psychosocial stressor, the psychological features of the participants play a role and could provide insights in this regard. Since weight is a main concern in P_AN_, this variable was considered too. Therefore, the following additional analyses were conducted. The effects of BMI, ED-symptomatology (EDI), psychological burden (BDI, GSI), chronic (TICS) and appraised stress (PASA-SI) on the HR and HRV response were estimated by means of a two-way ANCOVA for repeated measures 2 × 2 × 8. Subsequently, *post-hoc*-tests (i.e., estimated marginal means and bonferroni adjusted pairwise comparisons) were computed to specify differences throughout the measurement timepoints of relevant parameters. Discrepancies in subjective stress appraisal (PASA-SI) before and after stress exposure were calculated with ANOVAs for repeated measures. Differences in other psychological scales were analyzed by *t*-tests. Pearson-Product-Moment correlations were computed to determine the relationship between chronic stress (TICS), stress appraisal (PASA), BMI, and psychological burden (i.e., GSI, BDI, EDI) in our sample of P_AN_. The significance level was defined‘as *p* < 0.05.

## Results

### Psychological Measures

A summary of the sociodemographic variables of all study participants is displayed in Table 2. P_AN_ and HC were successfully matched for age and gender. Significant differences between the groups were identified in: BMI, sleeping problems and chronic stress. Additionally, P_AN_ showed highly pronounced values in psychological distress (GSI), depression (BDI) and ED symptomatology (EDI; Table 3). Since some of the P_AN_ had recovered some weight (BMI = 17.5–18.7 Kg/m^2^), while others were still underweight (BMI < 17.5 Kg/m^2^) we re-analyzed data comparing the two groups in terms of psychological measures, HR and HRV parameters. The analyses indicated comparable results (Table 3).

**Table 3:** Psychological variables and HRV-Reactivity in Patients with Anorexia Nervosa.

**P_**AN**_**	**Present scores*M (SD)***	**Norm values**			**Interpretation**	
BDI	42.46 (27.39)	<14 = normal 14–19 = mild 20–28 = moderate 29–63 = severe depression			Severe depression	
SCL (GSI)	12.57 (8.13)	Score 12 = ^*^Percentile 93%			Pronounced psychological burden	
EDI	148.94 (61.47)	Score 140–155 = ^*^Percentile = 85%			Pronounced ED-symptoms	
**P**_AN_ **≤** **17.5 vs. P**_AN_ **≥** **17.5 BMI** (**Kg/m**^2^)	^**(a)**^***U***	***Z***	***p***	η^2^		
BDI	28.50	−0.906	0.365	0.121		
SCL (GSI)	39.00	−0.414	0.679	0.032		
EDI	38.50	−0.454	0.650	0.031		
TICS	41.00	−0.248	0.840	0.023		
PASA–SI	43.49	−0.040	0.970	0.017		
VAS–TSST	43.50	−0.041	0.967	0.011		
**HRV-reactivity**	Condition^*^time^*^group				**Group**	
	***F (df)***	***p***	***η**^2^*	***F (df)***	***p***	***η**^2^*
HR	0.670 (3.85, 65)	0.611	0.038	0.193(1,17)	0.666	0.011
SDNN	1.69 (2.11,36.01)	0.196	0.091	0.079 (1,17)	0.78 1	0.005
RMSSD	230 (2.03., 34.64)	0.790	0.013	1.98 (1,17)	0.177	0.104
HF	0.140 (2.57, 36.09)	0.87	0.009	0.98 (1,17)	0.333	0.055
LF	0.730 (2.28, 32.57)	0.484	0.041	0.073 (1,17)	0.790	0.004
LF/HF	0.158 (1.69, 28.76)	0.85	0.009	0.194 (1,17)	0.669	0.011

*P_AN_, All patients with anorexia nervosa; HRV, Heart Rate Variability; BDI, Beck Depression Inventory; SCL, Symptom-Checklist-GSI, Global Severity Index; EDI, Eating Disorder Inventory; TICS, Trier Inventory Chronic Stress; PASA-SI, Primary Appraisal Secondary Appraisal - Stress Index*.

* *P_AN_ scored higher than 80/90% of the norm group. (a) Mann-Whitney-Test*.

#### Stress Perception und Appraisal

Acute stress perception (VAS) was comparable in both groups [*F*_(1_, _36)_ = 2.95, *p* < 0.094, η^2^ = 0.07], expressing significantly higher values after stress exposure, which indicated a successful stress induction [*F*_(1_, _36)_ = 93.03, *p* < 0.000, η^2^ = 0.72]. In contrast, stress appraisal (PASA-SI) was sig. higher in P_AN_ than in HC [*F*_(1_, _34)_ = 13.22, *p* < 0.001, η^2^ = 0.28]. PASA-subscales indicated differences in: *Threat* [*t*_(36)_ = 2.166, *p* = 0.037], *Self-Concept t*_(36)_ = −3.625, *p* = 0.001), and *Secondary Appraisal* (*t*_(36)_ = −3.171; *p* = 0.003).

Chronic and appraised stress were correlated with psychological variables. PASA-SI and EDI *r*(19) = 0.500, *p* = 0.040, but not between PASA-SI and BMI, BDI, or GSI. TICS and BDI, *r*(19) = 0.540, *p* = 0.021, GSI *r*(19) = 0.722, *p* = 0.000, EDI *r*(19) = 0.644, *p* = 0.003, but not between TICS and BMI.

#### Psychological Symptoms and Stress on the ANS Response

The effects of the ED-symptoms, psychological burden (BDI, GSI), appraised (PASA-SI), and chronic stress (TICS) on the HR and HRV response were estimated for each HRV-parameter by means of a two-way ANCOVA for repeated measures. Only stress appraisal and BMI affected ANS-reactivity. Significant effects were found in two parameters: SDNN; LF/HF-Ratio. **Perceived stress** on **SDNN** = Condition^*^time^*^PASA-SI *F*_(4.12, 140.15)_ = 2.676, *p* = 0.033, η^2^ = 0.073. Further analyses suggested that SDNN-HRV was distinctively low during stress and high at rest in individuals with a less pronounced stress appraisal (HC), this variability was less visible in participants with a higher stress appraisal (P_AN_). **BMI** on **LF/HF-Ratio** = Condition^*^time^*^BMI = *F*_(3.53, 60.16)_ = 3.339, *p* = 0.019, η^2^ = 0.164. Additional analyses indicated that participants with a normal BMI (21–25 kg/m^2^), in this case HC, clearly displayed a higher ratio during stress and a lower during rest. This pattern becomes less pronounced at a lower BMI, exhibiting an overall reduced ratio (P_AN_). Overall, no further sig. effects were shown (*F*s < 1, *p* = 0.080, η^2^ = 0.110).

### HRV Parameters

All HRV-data were tested for normal distribution with Kolmogorov–Smirnov test (K–S test). All HRV-parameters illustrated a skewed distribution (except for HR and SDNN) and were log-transformed for subsequent statistical analyses. The values and analyses of HR and HRV derived parameters are displayed in Tables 4, 5a,b.

Table 4HRV Parameters in P_AN_ and HC – ANOVA results.

**Table:** 

	**Condition**			**Group**			**Time**		
	***F (df)***	***p***	***η**^2^*	***F (df)***	***p***	***η**^2^*	***F (df)***	***p***	***η**^2^*
**HRV**									
HR	49.351 (1, 36)	0.000***	0.535	0.002 (1, 36)	0.968	0.000***	10.621 (2.09, 75.54)	0.000***	0.228
SDNN	5.357 (1, 36)	0.027	0.133	0.006 (1, 36)	0.938	0.000***	2.965 (4.83, 169.10)	0.015	0.078
RMSSD	1.335 (1, 36)	0.256	0.036	0.138 (1, 36)	0.712	0.004	6.971 (3.76, 135.54)	0.000***	0.162
HF	0.279 (1, 36)	0.601	0.008	0.054 (1, 36)	0.817	0.002	5.863 (4.61, 166.21)	0.000***	0.140
LF	8.361 (1, 36)	0.006	0.188	0.181 (1, 36)	0.673	0.005	7.835 (3.89, 140.23)	0.000***	0.179
LF/HF	9.345 (1, 36)	0.004	0.206	0.277 (1, 36)	0.602	0.008	7.178 (3.39, 122.25)	0.000***	0.166

**Table:** 

	Condition*Group			Time*Group			Condition*Time			Condition*Time*Group		
	*F (df)*	*p*	*η^2^*	*F*	*p*	*η^2^*	*F(df)*	*p*	*η^2^*	*F(df)*	*p*	*η^2^*
**HRV**												
HR	1.323 (1, 36)	0.258	0.035	3.725 (2.09, 75.54)	0.000***	0.244	46.847 (2.91, 104.98)	0.000***	0.565	9.326 (2.91, 104.98)	0.000***	0.206
SDNN	3.514 (1, 36)	0.069	0.091	0.871 (4.83, 169.10)	0.530	0.024	5.134 (4.11, 144.12)	0.001	0.128	2.109 (4.11, 144.12)	0.081	0.057
RMSSD	0.033 (1, 36)	0.858	0.001**	1.607 (3.76, 135.54)	0.179	0.043	16.461 (3.68, 132.62)	0.000***	0.314	3.869 (3.68, 132.62)	0.007	0.097
HF	0.563 (1, 36)	0.45	0.201	2.50 (4.61, 166, 21)	0.037	0.065	12.02 (4.21, 151.69)	0.000***	0.250	2.363 (4.21, 151.69)	0.052	0.062
LF	3.708 (1, 36)	0.062	0.093	2.536 (3.89, 140.23)	0.044	0.066	16.984 (4.50, 162.10)	0.000***	0.321	3.227 (4.50, 162.10)	0.011	0.082
LF/HF	0.317 (1, 36)	0.577	0.009	1.268 (3.39, 122.25)	0.266	0.034	3.382 (3.63, 130.94)	0.014	0.086	0.523 (3.63, 130.94)	0.702	0.014

P_AN_, Participants with Anorexia Nervosa; HC, Healthy Controls; M, Mean; SD, Standard Deviation; HRV, Heart Rate Variability. HR, Heart Rate; SDNN, standard deviation of NN intervals. RMSSD, Root mean square successive differences, HF, high frequency; LF, low frequency; LF/HF, Low frequency/ high frequency ratio, p ≤ 0.05*; p ≤ 0.01**; p ≤ 0.001***.

**Table 5a:** HRV parameters in the resting condition.

		**HR**				**LF**				**HF**				**LF/HF**				**RMSSD**				**SDNN**			
		***M***	***SD***	***t***	***p***	***M***	***SD***	***t***	***p***	***M***	***SD***	***t***	***p***	***M***	***SD***	***t***	***p***	***M***	***SD***	***t***	***p***	***M***	***SD***	***t***	***p***
**1**	P_AN_	80.81	(11.61)	1.066	0.294	3.23	(0.51)	−2.043	0.048	2.77	(0.60)	−0.646	0.522	2.45	(0.59)	−1.110	0.274	1.66	(0.264)	−0.761	0.451	71.55	(26.18)	−1.538	0.133
	HC	76.82	(11.48)			3.57	(0.50)			2.90	(0.65)			2.66	(0.55)			1.73	(0.31)			86.33	(32.66)		
**2**	P_AN_	79.89	(12.64)	0.770	0.447	3.07	(0.41)	−0.603	0.551	2.72	(0.50)	−0.643	0.524	2.34	(0.33)	0.389	0.700	1.61	(0.29)	−0.563	0.577	80.56	(62.95)	1.223	0.229
	HC	76.87	(11.54)			3.14	(0.33)			2.84	(0.64)			2.29	(0.41)			1.67	(0.33)			62.28	(16.79)		
**3**	P_AN_	78.18	(12.33)	1.007	0.321	3.06	(0.48)	−0.727	0.472	2.78	(0.63)	−0.532	0.598	2.27	(0.33)	0.032	0.975	1.61	(0.29)	−0.567	0.575	81.06	(65.21)	1.001	0.323
	HC	74.53	(9.83)			3.16	(0.43)			2.89	(0.67)			2.26	(0.45)			1.67	(0.33)			64.92	(26.215)		
**4**	P_AN_	78.58	(11.66)	1.177	0.247	2.90	(0.44)	−1.152	0.257	2.67	(0.60)	−1.073	0.290	2.23	(0.33)	0.399	0.692	1.60	(0.29)	−0.864	0.393	55.55	(24.44)	−0.423	0.675
	HC	74.53	(9.41)			3.06	(0.42)			2.88	(0.60)			2.18	(0.40)			1.68	(0.32)			58.74	(21.94)		
**5**	P_AN_	81.14	(13.40)	−0.123	0.903	2.97	(0.50)	0.670	0.507	2.61	(0.69)	0.571	0.572	2.35	(0.32)	−0.233	0.817	1.58	(0.36)	0.740	0.464	63.24	(44.64)	1.507	0.141
	HC	81.60	(9.41)			2.87	(0.39)			2.49	(0.65)			2.38	(0.40)			1.50	(0.32)			47.41	(10.19)		
**6**	P_AN_	79.00	(9.54)	1.471	0.150	3.06	(0.50)	−0.496	0.623	2.75	(0.64)	−0.379	0.707	2.31	(0.33)	0.052	0.959	1.61	(0.31)	−0.504	0.617	61.64	(33.93)	0.295	0.769
	HC	74.74	(8.22)			3.13	(0.40)			2.87	(0.65)			2.30	(0.35)			1.67	(0.33)			59.12	(15.25)		
**7**	P_AN_	78.43	(10.28)	0.811	0.423	3.05	(0.55)	−0.034	0.973	2.81	(0.68)	0.147	0.884	2.23	(0.31)	−0.730	0.470	1.64	(0.33)	−0.632	0.531	64.75	(36.47)	0.983	0.332
	HC	75.93	(8.71)			3.05	(0.35)			2.78	(0.66)			2.32	(0.43)			1.73	(0.44)			55.92	(14.29)		
**8**	P_AN_	78.04	(11.54)	0.118	0.906	3.03	(0.54)	0.348	0.730	2.74	(0.62)	−0.494	0.624	2.28	(0.28)	0.846	0.403	1.62	(0.33)	−0.783	0.439	60.01	(27.54)	0.786	0.437
	HC	77.64	(8.87)			2.97	(0.42)			2.84	(0.68)			2.18	(0.47)			1.73	(0.47)			54.51	(13.17)		

*Measurement timepoints =1 (Baseline), 2 (Rest), 3 (Rest), 4 (Rest), 5–8 (Recovery Phase). P_AN_, Participants with Anorexia Nervosa. HC, Healthy Controls; Log-transformed values (except HR and SDNN); HR, Heart Rate; LF, Low Frequency; HF, High Frequency; LF/HF-Ratio; RMSSD, Root Mean Square Standard Deviation; SDNN, Standard Deviation Normal to Normal*.

**Table 5b:** HRV parameters in the stress condition.

		**HR**				**LF**				**HF**				**LF/HF**				**RMSSD**				**SDNN**			
**HR**		***M***	***SD***	***t***	***p***	***M***	***SD***	***t***	***p***	***M***	***SD***	***t***	***p***	***M***	***SD***	***t***	***p***	***M***	***SD***	***t***	***p***	***M***	***SD***	***t***	***p***
1	P_AN_	78.87	(13.80)	0.494	0.624	3.31	(0.60)	−1.884	0.068	2.79	(0.65)	−1.022	0.314	2.51	(0.59)	−0.748	0.459	1.66	(0.33)	−1.191	0.241	77.56	(34.56)	−1.576	0.120
	HC	76.92	(10.31)			3.65	(0.50)			2.99	(0.55)			2.65	(0.53)			1.78	(0.30)			94.16	(30.17)		
2	P_AN_	83.20	(11.38)	1.481	0.147	2.95	(0.40)	−2.359	0.024	2.57	(0.50)	−1.892	0.067	2.38	(0.26)	0.533	0.597	1.57	(0.28)	−1.528	0.135	66.33	(32.55)	−335	0.739
	HC	78.33	(8.66)			3.22	(0.29)			2.89	(0.54)			2.32	(0.34)			1.72	(0.28)			69.34	(21.74)		
3	P_AN_	89.15	(13.07)	−2.802	0.008	2.98	(0.42)	0.908	0.370	2.59	(0.61)	1.180	0.246	2.39	(0.34)	−1.098	0.279	1.50	(0.31)	1.339	0.189	64.51	(24.43)	0.438	0.664
	HC	104.04	(19.11)			2.87	(0.35)			2.35	(0.63)			2.51	(0.35)			1.37	(0.30)			61.25	(21.35)		
4	P_AN_	88.14	(13.88)	−2.633	0.012	2.98	(0.52)	1.450	0.156	2.46	(0.63)	1.291	0.205	2.521	(0.29)	−0.317	0.753	1.44	(0.29)	1.770	0.085	58.05	(27.57)	1.662	0.106
	HC	102.83	(19.97)			2.75	(0.48)			2.20	(0.62)			2.54	(0.20)			1.26	(0.33)			45.11	(19.79)		
5	P_AN_	81.86	(15.03)	−0.492	0.625	2.97	(0.50)	0.670	0.507	2.61	(0.69)	0.571	0.572	2.35	(0.32)	−0.233	0.817	1.58	(0.36)	0.740	0.464	70.64	(67.98)	1.331	0.198
	HC	83.97	(11.13)			2.87	(0.39)			2.49	(0.65)			2.38	(0.40)			1.50	(0.32)			49.10	(18.76)		
6	P_AN_	75.04	(15.39)	−0.453	0.654	3.06	(0.50)	−0.496	0.623	2.75	(0.64)	−0.379	0.707	2.31	(0.33)	0.052	0.959	1.61	(0.31)	−0.504	0.617	73.49	(47.50)	0.624	0.538
	HC	77.01	(10.94)			3.13	(0.40)			2.83	(0.65)			2.30	(0.35)			1.67	(0.33)			66.00	(22.00)		
7	P_AN_	74.85	(13.98)	0.162	0.872	3.05	(0.55)	−0.034	0.973	2.81	(0.68)	0.147	0.884	2.23	(0.31)	−0.730	0.470	1.64	(0.33)	−0.632	0.532	81.64	(78.10)	1.189	0.243
	HC	73.92	(20.86)			3.05	(0.35)			2.78	(0.66)			2.32	(0.43)			1.73	(0.44)			59.12	(19.27)		
8	P_AN_	76.37	(12.05)	0.423	0.675	3.03	(0.54)	0.348	0.730	2.74	(0.62)	−0.494	0.624	2.28	(0.28)	0.846	0.403	1.62	(0.33)	−0.783	0.439	69.68	(44.72)	1.189	0.228
	HC	74.00	(21.18)			2.97	(0.42)			2.84	(0.68)			2.18	(0.47)			1.73	(0.47)			55.64	(19.47)		

*Measurement timepoints =1 (Baseline), 2 (Preparation), 3 (Before public speaking), 4 (After math task), 5–8 (Recovery Phase). P_AN_, Participants with Anorexia Nervosa; HC, Healthy Controls; Log-transformed values (except HR and SDNN). HR, Heart Rate; LF, Low Frequency; HF, High Frequency; LF/HF-Ratio. RMSSD, Root Mean Square Standard Deviation; SDNN, Standard Deviation Normal to Normal*.

**Figure 2:**
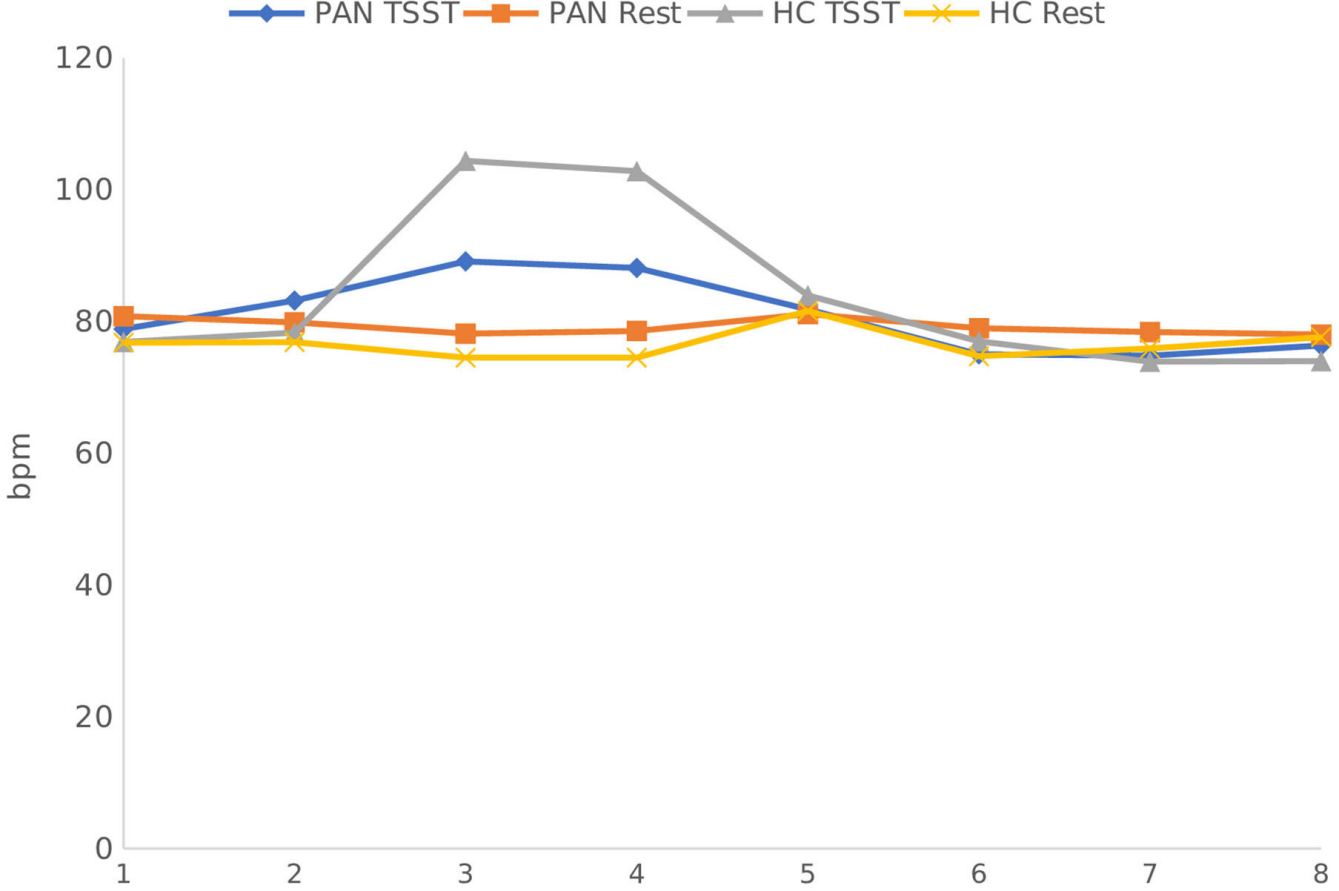
Heart Rate Response in PAN and HC during stress and at rest. Measurement timepoints = 1 (Baseline), 2 (Preparation), 3 (Before public speaking), 4 (After math task), 5-8 (Recovery Phase). The measurements in the control condition were collected on equivalent timepoints.

**Figure 3:**
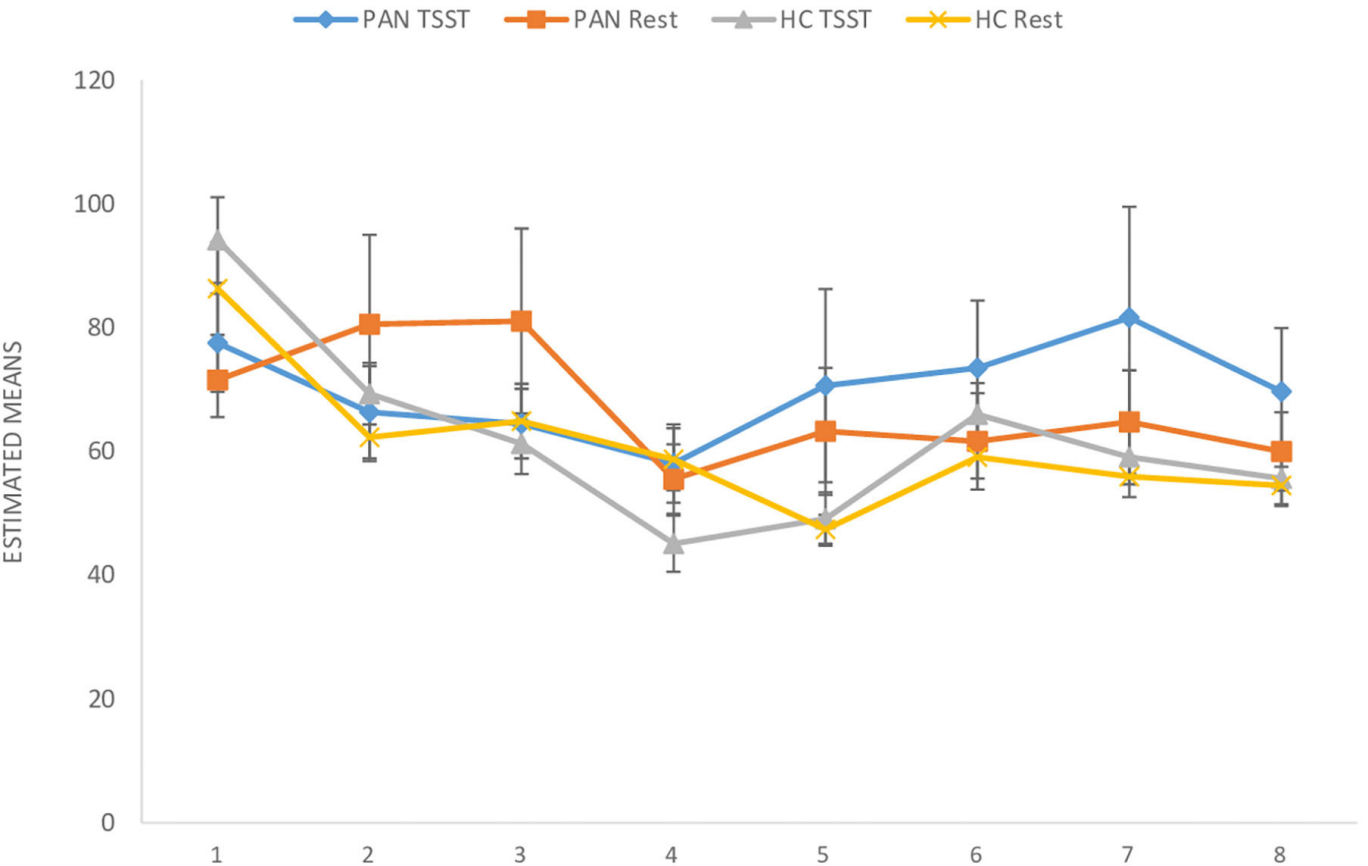
SDNN (ms) in PAN and HC during stress and at rest. SDNN, Standard Deviation Normal to Normal. Measurement timepoints = 1 (Baseline), 2 (Preparation), 3 (Before public speaking), 4 (After math task), 5-8 (Recovery Phase). The measurements in the control condition were collected on equivalent timepoints.

**Figure 4:**
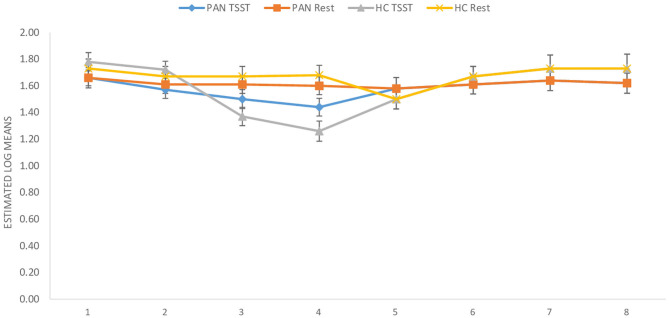
RMSSD (ms) in PAN and HC during stress and at rest. RMSSD Root Mean Square Standard Deviation. Measurement timepoints = 1 (Baseline), 2 (Preparation), 3 (Before public speaking), 4 (After math task), 5-8 (Recovery Phase). The measurements in the control condition were collected on equivalent timepoints.

**Figure 5:**
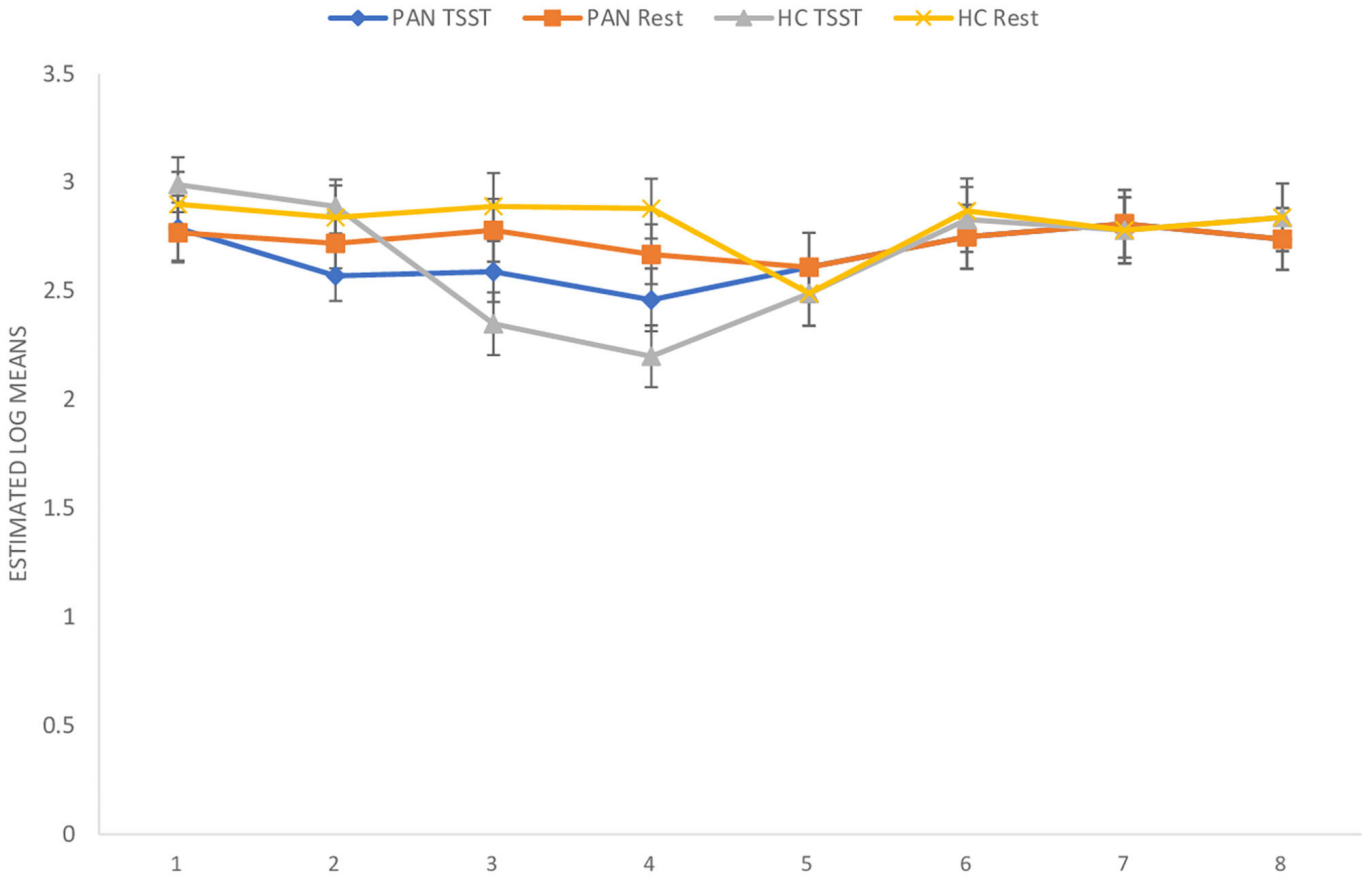
HF (ms2) in PAN and HC during stress and at rest. HF, High Frequency. Measurement timepoints = 1 (Baseline), 2 (Preparation), 3 (Before public speaking), 4 (After math task), 5-8 (Recovery Phase). The measurements in the control condition were collected on equivalent timepoints.

**Figure 6:**
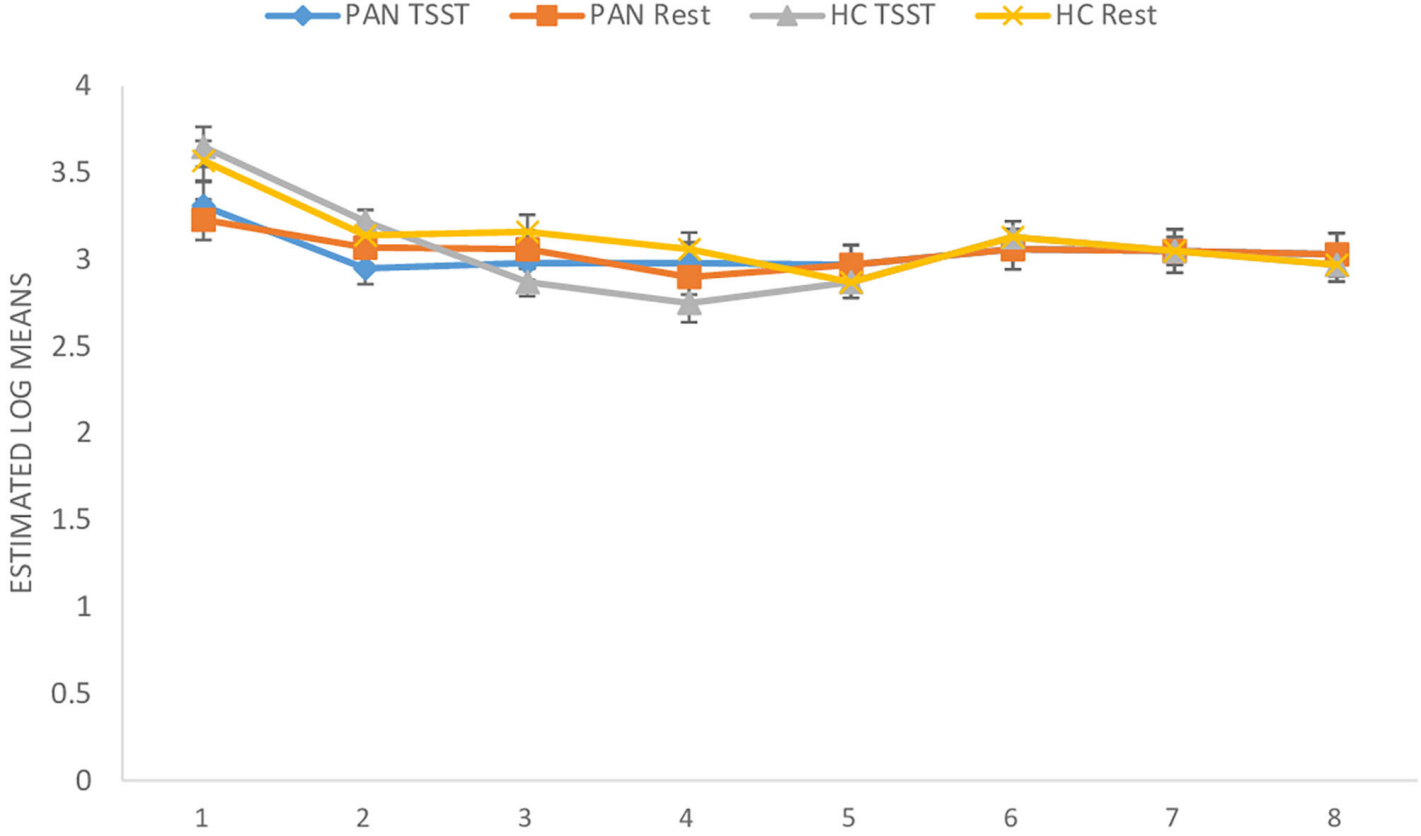
LF (ms2) in PAN and HC during stress and at rest. LF, Low Frequency. Measurement timepoints = 1 (Baseline), 2 (Preparation), 3 (Before public speaking), 4 (After math task), 5-8 (Recovery Phase). The measurements in the control condition were collected on equivalent timepoints.

**Figure 7:**
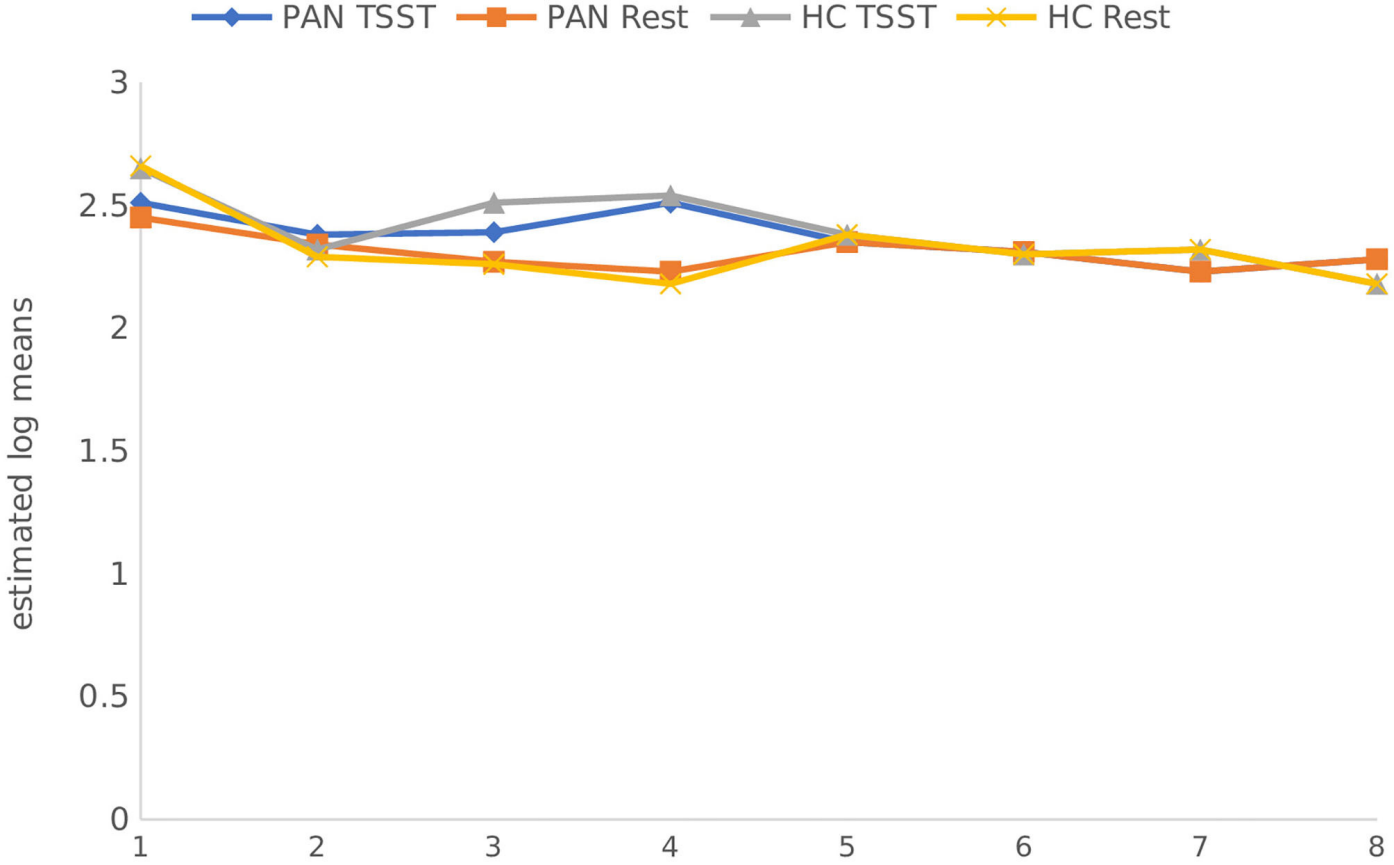
LF/HF-Ratio (ms^2^) in P_AN_ and HC during stress and at rest. LF/HF-Ratio, Low Frequency/High Frequency Ratio. Measurement timepoints = 1 (Baseline), 2 (Preparation), 3 (Before public speaking), 4 (After math task), 5–8 (Recovery Phase). The measurements in the control condition were collected on equivalent timepoints.

**(H**_1_**)**. HR values (at rest) were comparable between P_AN_ and HC (Tables 4, 5a,b). **(H**_2_**)**. In terms of HRV tone, a significance was shown in LF—with P_AN_ showing lower values than controls (Tables 4, 5a,b). The remaining parameters were comparable between groups, PNS hyperactivity was not observed in P_AN_ at rest (Tables 4, 5a,b). (**H**_3_). Only a sig. effect of condition^*^time^*^group was shown. P_AN_ clearly illustrated a blunted HR response in comparison to the HC (see Tables 4, 5a,b; Figure 2). (**H**_4_). In terms of group differences in ANS reactivity, the following patterns were revealed: **SDNN**. Only a significant effect of *time* and *condition* and an interaction effect: condition^*^time were shown, indicating increased values during stress than at rest in all participants over time (Tables 4, 5a,b; Figure 3). At a descriptive level, a weaker reactivity was visible in P_AN_ relative to the HC (see Table 5b). **RMSSD**. A significant effect of *time* and two sig. interaction effects were observed: (1) Condition^*^time (2) Condition^*^time^*^group. P_AN_ showed an attenuated stress response compared to the HC. No further sig. effects were demonstrated (Tables 4, 5a,b; Figure 4). **HF**. A sig. effect of *time* and two sig. interaction effects were found: (1) Condition^*^time and (2) Time^*^group. Overall, there were sig. differences in HF-values, with P_AN_ recording higher values than HC over time. The interaction effect of condition^*^time^*^group was not significant (Tables 4, 5a,b, Figure 5). **LF**. A sig. effect of *condition* and *time* and a multiple sig. *interaction* effects were evinced: (1) Time^*^group, (2) condition^*^time, (3) condition^*^time^*^group, indicating a lower HRV-LF-reactivity in patients, compared to HC (Tables 4, 5a,b, Figure 6). **LF/HF-Ratio**. Only a highly sig. effect of *condition* and *time* and a sig. *interaction* effect of condition^*^time were revealed: All participants displayed a higher ratio over time during stress than at rest (Tables 4, 5a,b, Figure 7). **(H**_5_**)**. In terms of HRV reactivity, P_AN_ exhibited a reduced SNS reactivity, without PNS dominance (see Tables 4, 5a,b). Overall, large to medium effect sizes were revealed (see Table 4).

## Discussion

Measuring HRV as a proxy to assess ANS reactivity has many advantages and allows a detailed analysis of the SNS and PNS contribution. In addition, HRV reflects the capacity of the body to deal with stress and predicts psychological and physiological health. Investigating the HRV activity and its alterations might aid the development of novel ways to target AN-symptomatology and reduce mortality rates. For this purpose, we extensively investigated the ANS reactivity to a psychosocial stressor in P_AN_ and a matched sample of HC under highly standardized laboratory conditions and analyzed different HRV-parameters. In sum, the stress induction was successful (VAS). Compared to HC, PAN appraised (PASA) the experimental procedures as more threatening and experienced themselves as less influential over the ongoing circumstances. Further, they felt less capable of coping with the given situation than healthy adults. Moreover, chronic and appraised stress were more strongly associated with psychological symptoms than with weight. Concerning reactivity, stress appraisal and weight significantly affected the ANS response (i.e., SDNN; LF/HF-Ratio) with P_AN_ showing a reduced ratio overall, relative to the HC. In this regard, underweight and weight recovered patients were comparable in terms of cardiovascular reactivity and psychological measures. Their BMI variations were not big enough to differently affect reactivity.

Concerning HR and HRV, our data revealed similarities between the groups in almost all parameters during resting conditions. A significant difference was only shown in LF-HRV, indicating a decreased baroreflex activity in patients, compared to controls. In terms of HRV-reactivity: Compared to HC, patients clearly evinced a blunted HR response and displayed an attenuated PNS reactivity, as indicated by a less pronounced decline in RMSSD and HF-HRV (see Figures 4, 5). Relative to the control group, patients also demonstrated an attenuated SNS reactivity to stress, as indexed by a smaller increase in LF-HRV (Figure 6). Overall, patients and controls showed a similar general HRV reactivity as indicated by LF/HF-ratio and SDNN. Both groups presented sympathetic dominance during stress, with a slightly weaker SNS-reactivity in P_AN_–but only at a descriptive level.

In general, the outcomes of the present study extended previous research and provided new data specific to P_AN_ concerning their ANS response in the context of psychosocial stress. **(H**_1_**)** Contrary to our first hypothesis, our data indicated a similar tonic HR between P_AN_ and HC. This is in line with previous results reporting comparable baseline HR-values between both groups (Zonnevylle-Bender et al., 2005; Monteleone et al., 2011; Het et al., 2020). Nevertheless, our findings contradicted those of Het et al. (2015), who found a significantly lower HR (baseline) in ED-patients than in healthy participants. The discrepancy between our findings and those of Het et al. (2015) can be explained by the quality of the patient sample. The patients in their study were recruited immediately after admission to a treatment clinic, implying an acute ED-phase, since they just started treatment. It is known that bradycardia is a common symptom in the acute phase of AN (Yoshida et al., 2006; Mazurak et al., 2011). In contrast, the participants in our sample were recruited after a stabilization phase during an inpatient treatment, showing a higher (mean) BMI than in the mentioned sample. **(H**_2_**)** Further, we assumed parasympathetic hyperactivity and a decreased SNS activity in P_AN_ at rest, compared to the HC. Our results confirmed the latter, expressing lower HRV-LF in P_AN_, as previously described (Mazurak et al., 2011; Jacoangeli et al., 2013; Bomba et al., 2014). Nevertheless, parasympathetic hyperactivity in P_AN_ could not be observed, since other HRV-parameters (HF, LF/HF, RMSSD, SDNN) were comparable between P_AN_ and HC. Hence, our second hypothesis only received partial support. This inconsistency between our result an those previously mentioned (Mazurak et al., 2011; Jacoangeli et al., 2013; Bomba et al., 2014) are probably due to differences in the patient population. For instance, these studies included P_AN_ with an average BMI between 12.9 and 17.7, or BMI ≤ 15, implying emaciating. On the other hand, the P_AN_ we evaluated were being treated and had recovered some weight. Therefore, it is plausible to assume that some irregularities in cardiovascular activity were restored, as previously shown in weight recovered patients (Miller et al., 2009; Het et al., 2020). Even so, abnormalities in HR and HRV reactivity were still observed in P_AN_, as previously hypothesized (H_3_, _4_): We expected a blunted HR response **(H**_3_**)** and a low HRV reactivity **(H**_4_**)** in P_AN_ compared to HC. Our data confirmed both, replicating past evidence (e.g., Monteleone et al., 2011; Het et al., 2015, Monteleone et al., 2018; Het et al., 2020). Our last hypothesis **(H**_5_**)** confirmed a reduced SNS reactivity in P_AN_. However, a parasympathetic hyperreactivity could not be replicated. The former finding coincided with past studies reporting an attenuated sympathetic response to the TSST in patients (Monteleone et al., 2011; Het et al., 2015). Our latter finding of a parasympathetic inhibition in response to the TSST suggested an inadequate parasympathetic regulation, indicating difficulties in P_AN_ to downregulate stress (Carney et al., 2005). This outcome fit the results of Rommel et al. (2015), who recorded a similar response pattern in a sample of P_AN_ (SNS activity was not investigated), but contradicted the findings of Het et al. (2015) and Het et al. (2020). They rather observed parasympathetic dominance in their patient sample during early treatment (2015), and a normal PNS reactivity after treatment (2020). A plausible explanation might lie in the treatment status and sample composition, considering that our patient sample was in the middle of therapy and included only participants with P_AN_, rather than an ED-mixed group.

In short: compared to HC, the patients in our study demonstrated hyporeactivity in both, SNS and PNS. This makes sense, since SNS is associated with dieting and chronic stress as indicated by pronounced values in TICS and marked ED-symptomatology (EDI) in our participants. On the other hand, PNS hyporeactivity is associated with depression (Jarrett et al., 2003), as indexed by elevated BDI-scores in our P_AN_ sample. PNS hyperactivity is related to bradycardia and is mostly characteristic in the acute-phase of AN (as an adaptive mechanism; Roche et al., 2004; Yoshida et al., 2006) which was not observed in our sample. Consequently, it can be implied that ANS functionally was partially restored.

Ultimately, these findings provide strong evidence for a hyporesponsiveness of the ANS in P_AN_. Overall, it was shown that abnormal ANS activity is partly reversed with weight recovery and treatment, but irregularities in reactivity persisted despite these changes. Specifically, (increased) stress appraisal and (low) weight significantly affected HRV reactivity (i.e., blunted response). In average, patients experienced stress as threatening and felt less skillful to cope with it. These outcomes are a major strength of the present study considering that HRV reactivity in the context of psychosocial stress has not been studied before in samples specific to P_AN_. Many researchers who have conducted comparable experiments with ED-mixed samples pointed out the need of ED-specific findings, since there exist marked differences in emotional and physiological patterns (e.g., Peschel et al., 2016) that cannot be clearly identified if studied as a group. Further, our study offers an extensive analysis of the ANS reactivity in P_AN_, which fosters understanding in the psychological and physiological mechanisms of their ANS response. At the same time, it provides input for potential treatment designs, e.g., HRV-Biofeedback training (Scolnick et al., 2014). Importantly, it suggests that ANS imbalances can manifest differently according to the stage of illness, treatment and weight status, as observed in different patient samples. A further strength of our research were the highly standardized context and laboratory setting: Homogenous samples of HC and patients due to our strict inclusion and exclusion criteria (which is pivotal for minimizing the influence of confounding variables). In addition, participants were perfectly age and gender matched. Moreover, we implemented a highly standardized stressor (TSST), which reliably activates the ANS. Notwithstanding, our study hosts some limitations. Regardless of the significant effects reported, an evident limitation is the small sample size in our study. Also, the generalizability of our findings is limited to female patients. Therefore, larger studies with more robust samples are needed to back up the results and to shed light on to the non-significant trends. A further limitation is that we were not able to collect data related to illness and treatment duration (e.g., number of days at the clinic before participation). Future research would benefit from including this data in future analyses. Thereupon, it remains to clarify whether ANS reactivity can be completely restored, not only after weight recovery, but specially after improving well-being and coping skills (e.g., stress appraisal). Additionally, further experiments may study the effects of HRV-biofeedback interventions on subjective stress appraisal and well-being. In conclusion, these findings suggested ANS hyporeactivity in P_AN_, despite normalized basal activity. Specifically, it was revealed that ANS reactivity was most affected by heightened stress appraisal and low weight, rather than other psychological symptoms (e.g., general ED-symptomatology). Therefore, we supposed that besides weight recovery, improvement in stress appraisal would be beneficial. Consequently, it is recommended to help patients improve stress appraisal by developing and enhancing their coping and social skills. In addition, exercise sessions with biofeedback training might support cardiac health and HRV-regulation. A combination of both may improve treatment outcomes and regulate stress reactivity.

## Data Availability Statement

The datasets presented in this article are available from the corresponding author, on reasonable request.

## Ethics Statement

The studies involving human participants were reviewed and approved by Ethics Committee of the Medical faculty of the Technical University of Dresden, Germany (No#EK25012013). The patients/participants provided their written informed consent to participate in this study.

## Author Contributions

KP and SW: conceptualization. BS and IS: methodology. KP and SW: software. HB, BS, and BH: validation. IS: formal analysis, writing—original draft preparation, visualization, and writing—review and editing. SP: investigation. SR: resources. BH: data curation. KP and HB: supervision. KP: project administration. All authors contributed to the article and approved the submitted version.

## Conflict of Interest

The authors declare that the research was conducted in the absence of any commercial or financial relationships that could be construed as a potential conflict of interest.
